# Efficacy and safety of eltrombopag in Japanese patients with chronic liver disease and thrombocytopenia: a randomized, open-label, phase II study

**DOI:** 10.1007/s00535-012-0600-5

**Published:** 2012-06-08

**Authors:** Takumi Kawaguchi, Atsumasa Komori, Masataka Seike, Shigetoshi Fujiyama, Hiroshi Watanabe, Masatoshi Tanaka, Shotaro Sakisaka, Makoto Nakamuta, Yutaka Sasaki, Makoto Oketani, Toshihiro Hattori, Koichi Katsura, Michio Sata

**Affiliations:** 2 Department of Digestive Disease Information & Research, and Department of Medicine, Kurume University School of Medicine, 67 Asahi-machi, Kurume, Fukuoka 830-0011 Japan; 3 Clinical Research Center, National Hospital Organization Nagasaki Medical Center, Nagasaki, Japan; 4 Department of Internal Medicine 1, Faculty of Medicine, Oita University, Oita, Japan; 5 Department of Gastroenterology and Hepatology, NTT West Kyushu Hospital, Kumamoto, Japan; 6 Hepatology Division, Fukuoka Red Cross Hospital, Fukuoka, Japan; 7 Department of Gastroenterology and Medicine, Kurume University Medical Center, Fukuoka, Japan; 8 Department of Gastroenterology and Medicine, Fukuoka University School of Medicine, Fukuoka, Japan; 9 Department of Gastroenterology, Clinical Research Center, Kyushu Medical Center, National Hospital Organization, Fukuoka, Japan; 10 Department of Gastroenterology and Hepatology, Graduate School of Medical Sciences Kumamoto University, Kumamoto, Japan; 11 Department of Digestive and Lifestyle-Related Diseases, Health Research Human and Environmental Sciences, Kagoshima University Graduate School of Medical and Dental Sciences, Kagoshima, Japan; 12 GlaxoSmithKline, Tokyo, Japan

**Keywords:** Thrombopoietin receptor agonist, Pharmacokinetics, Invasive procedures, Inter-ethnic difference

## Abstract

**Background:**

Eltrombopag is an oral thrombopoietin receptor agonist that stimulates thrombopoiesis and shows higher exposure in East Asian patients than in non-Asian patients. We evaluated the pharmacokinetics, efficacy, and safety of eltrombopag in Japanese patients with thrombocytopenia associated with chronic liver disease (CLD).

**Methods:**

Thirty-eight patients with CLD and thrombocytopenia (platelets <50,000/μL) were enrolled in this phase II, open-label, dose-ranging study that consisted of 2 parts. In the first part, 12 patients received 12.5 mg of eltrombopag once daily for 2 weeks. After the evaluation of safety, 26 patients were randomly assigned to receive either 25 or 37.5 mg of eltrombopag once daily for 2 weeks in the second part.

**Results:**

Pharmacokinetics showed that the geometric means of the maximum plasma concentration (*C*_max_) and the area under the curve (AUC) in the 12.5 mg group were 3,413 ng/mL and 65,236 ng h/mL, respectively. At week 2, the mean increases from baseline in platelet counts were 24,800, 54,000, and 60,000/μL in the 12.5, 25, and 37.5 mg groups, respectively. The median platelet counts increased within 2 weeks of the beginning of administration in all groups, and remained at the same level throughout the 2-week post-treatment period in the 12.5 mg group, whereas the platelet counts peaked a week after the last treatment in both the 25 and 37.5 mg groups. Most adverse events reported were grade 1 or 2; 2 patients in the 37.5 mg group had drug-related serious adverse events.

**Conclusions:**

Eltrombopag ameliorated thrombocytopenia in Japanese patients with CLD and thrombocytopenia. The recommended dose for these patients is 25 mg daily for 2 weeks.

## Introduction

Thrombocytopenia is frequently observed in patients with chronic liver disease (CLD) and is considered a surrogate marker for the severity of liver disease [1, 2]. Besides hypersplenism secondary to portal hypertension, decreased thrombopoietin (TPO) production by hepatocytes is an important cause of thrombocytopenia in this patient population [3].

Some invasive procedures, such as liver biopsy, radiofrequency ablation (RFA), and partial hepatectomy for hepatocellular carcinoma (HCC), are performed as part of the therapeutic management of patients with CLD. Thrombocytopenia often interferes with such invasive procedures and platelet transfusions may be required [4–9]. The frequency of splenectomy and platelet transfusions is significantly higher in HCC patients with severe thrombocytopenia (<50,000/μL) than in those without thrombocytopenia [10]. However, both splenectomy and platelet transfusions have limitations and disadvantages; for example, splenectomy is invasive and may be associated with life-threatening short- as well as long-term complications, and platelet transfusions are short-acting and may cause transfusion-related complications [11–14]. Thus, alternative therapeutic options to platelet transfusions or splenectomy would provide an important clinical benefit.

Eltrombopag (GlaxoSmithKline, Ware, UK) is an orally bioavailable, small-molecule, non-peptide thrombopoietin receptor (TPO-R) agonist, which has been approved for the treatment of chronic idiopathic thrombocytopenic purpura (ITP). Eltrombopag induces the proliferation and differentiation of megakaryocytes, resulting in an increase in platelet production in chimpanzees and humans [15]. Eltrombopag increases platelet counts in a dose-dependent fashion in patients with ITP and in those with thrombocytopenia with hepatitis C virus (HCV) infection, as well as in healthy volunteers [16–19].

Eltrombopag is primarily metabolized in the liver, and a higher plasma eltrombopag exposure has been reported in HCV-infected patients compared with ITP patients and healthy volunteers [20]. Furthermore, inter-ethnic differences in the pharmacokinetics of eltrombopag have been reported; the area under the curve (AUC) of eltrombopag in ITP patients and healthy volunteers was approximately 2-fold higher in East Asian subjects than in those of non-Asian origin [21]. Thus, the pharmacokinetics of eltrombopag in Japanese patients with CLD may be different from those previously reported in ITP patients and Caucasian patients [17–19].

The aim of this phase II study was to assess the efficacy and safety of eltrombopag in Japanese patients with CLD and thrombocytopenia using lower daily doses (12.5, 25, or 37.5 mg) than those typically used for Caucasian patients.

## Methods

### Patients

A total of 38 patients with CLD (25 with HCV infection, 7 with hepatitis B virus [HBV] infection, 1 with both HCV and HBV infections, and 5 with cryptogenic cirrhosis) were enrolled from 10 Japanese institutions between January and August 2009. Eligible patients were 20 years of age or older and had thrombocytopenia (baseline platelet counts <50,000/μL). Patients were also required to have a Child–Pugh score of 9 or less (Child–Pugh class A or B) and hemoglobin concentration of >8 g/dL for at least 4 weeks before enrollment. Platelet transfusions and interferon therapies had to be completed at least 2 and 4 weeks before enrollment, respectively.

Patients with evidence of human immunodeficiency virus (HIV) infection, evidence of portal vein thrombosis on abdominal imaging within 3 months before enrollment, a history of arterial or venous thrombosis with 2 or more thrombosis risk factors, or platelet agglutination abnormalities were excluded from the study. Patients with active World Health Organization (WHO) grade 3 or 4 bleeding were also excluded [22]. Women who were pregnant or breastfeeding were not eligible, nor were patients who required the use of polyvalent cation-containing medicines, which are known to form chelates with eltrombopag. Patients requiring medications that are known to affect platelet functions [e.g., aspirin, nonsteroidal anti-inflammatory drugs (NSAIDs), and anti-platelet agents], and patients requiring hydroxymethylglutaryl-CoA reductase inhibitors (for which exposure might be increased by eltrombopag administration) were also excluded.

Diagnosis of liver cirrhosis was assessed by an aspartate aminotransferase-to-platelet ratio index (APRI) of >1 [23] and an FIB4 index of >3.25 according to the Practice Guideline for Liver Cirrhosis edited by the Japanese Society of Gastroenterology [24]. Creatinine clearance was estimated by the Cockroft–Gault formula [25] in a post-hoc analysis.

This study was approved by each institutional review board and was conducted in accordance with the Declaration of Helsinki, Good Clinical Practice guidelines, and local laws and regulations. All patients provided written informed consent before enrollment.

### Treatments

Because inter-ethnic differences in the eltrombopag AUC have been found in earlier studies [21, 26], lower doses (12.5, 25, 37.5 mg) of eltrombopag were used than the doses (30, 50, 75 mg) used in a previous study conducted in cirrhotic patients (predominantly Caucasian) with HCV infection [18]. All doses were administered with the patients in a fasting state, in which the patients were required to refrain from food ingestion for at least 2 h pre- and post-dose.

### Study design and procedures

**Fig. 1:**
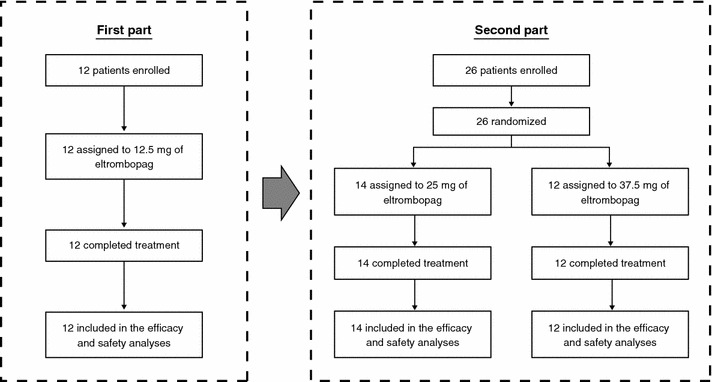
Study design. The study was a multicenter, open-label, dose-ranging phase II study that used a unique sequential design and consisted of 2 parts. After review, by a Safety Review Committee, of safety data from the 12.5 mg group (first part), new patients were randomly assigned to receive 25 or 37.5 mg of eltrombopag once daily for 2 weeks in the second part

This was a multicenter, open-label, dose-ranging phase II study that used a unique sequential design and consisted of 2 parts. In the first part, 12 patients received 12.5 mg of eltrombopag once daily for 2 weeks, and the data were reviewed by a Safety Review Committee (Fig. 1). After the evaluation of safety in the first part, in the second part 26 new patients were randomly allocated, at a 1: 1 ratio, to receive either 25 or 37.5 mg of eltrombopag once daily for 2 weeks. An additional week of treatment was allowed if platelet counts were <80,000/μL at week 2 (Fig. 1). Eltrombopag treatment was to be discontinued if platelet counts were >200,000/μL during the treatment period. Invasive procedures could be performed after the end of treatment with eltrombopag per the investigator’s decision. All patients were assessed for the efficacy and safety of eltrombopag every week during the treatment period, and at 4 days, 1 week, and 2 weeks post-treatment.

The primary endpoint of the study was the change from baseline in platelet counts at the end of week 2. Secondary endpoints were: (1) response rate (achieving platelet counts of ≥80,000/μL) of eltrombopag administered for 2 weeks, or after an additional week, (2) median platelet counts, and (3) safety. The severity of adverse events was graded using the Division of AIDS Table for Grading the Severity of Adult and Pediatric Adverse Events (version 1.9, dated December 2004). The effects of pretreatment with eltrombopag on the prevalence of perioperative bleeding and platelet transfusions were also evaluated when invasive procedures, such as liver biopsy, RFA, and partial hepatectomy, were performed after the end of treatment.

### Randomization and masking

In the second part of this study, patients were randomly allocated to either the 25 or 37.5 mg group. Randomization was centrally performed, and the random assignment was stratified according to baseline Child–Pugh class (A or B). This study was not blinded.

### Pharmacokinetics

Serial samples were collected pre-dose (prior to administration on day 14), and 1, 2, 4, 6, 8, 10, and 24 h post-dose in the 12.5 mg group. Pharmacokinetic parameters [maximum plasma concentration (*C*_max_) and time to maximum plasma concentration (*T*_max_), AUC_0–*t*_, and AUC_0–*τ*_] in the 12.5 mg group were determined with actual sample times using non-compartmental analysis and summary statistics. Sparse samples were collected in the 25 or 37.5 mg group following 1 of 2 schedules: (1) pre-dose (prior to administration on day 14) and 0.5–3, and 24 h post-dose, or (2) 4–6, 8–12, and 24 h post-dose. The geometric mean of the plasma eltrombopag concentration was summarized in each group. One patient each from the 12.5 and 37.5 mg groups was excluded from the summary statistics, as these 2 patients had used a cation-containing antacid.

### Statistical analyses

On the basis of a previous study [18], the changes from baseline in platelet counts at week 2 were assumed to be 30,000, 80,000, and 100,000/μL in the 12.5, 25, and 37.5 mg groups, respectively, and the standard deviation was 50,000/μL in each group. Based on Monte Carlo simulations, 12 evaluable patients per group were needed to provide 90 % or more power to detect a linear dose trend and saturation at a medium dose trend. No interim analysis was planned. Descriptive statistics and frequency tables were used to summarize demographics, baseline characteristics, and safety data. The analyses included all patients who had received at least one dose of eltrombopag.

The primary endpoint was analyzed using point estimates and 2-sided 95 % confidence intervals (CIs) by each group. In an exploratory analysis, the changes from baseline in platelet counts at week 2 were analyzed using analysis of covariance (ANCOVA), with baseline platelet counts as a covariate to detect the following dose response patterns: linearity in 3 doses, saturation at the medium dose (25 mg), or onset of response at the high dose (37.5 mg). A similar analysis, with both baseline platelet counts and Child–Pugh class as covariates, was conducted as a secondary analysis. This model used the changes in platelet counts of each patient. No adjustment for multiplicity was made because these analyses were exploratory. Other secondary endpoints were analyzed using point estimates and 2-sided 95 % CIs for each group. Analyses were based on the observed data.

This study is registered at ClinicalTrials.gov with identifier number: NCT00861601.

### Role of the funding source

The protocol was developed by the principal investigators and employees of the sponsor. Data were collected and analyzed by the sponsor. All authors had access to the primary data and vouch for the completeness and accuracy of the data and analyses. Interpretation of the data and decisions related to the content of the report were made through collaboration among all authors. The corresponding author had final responsibility for the decision to submit for publication.

## Results

### Patient characteristics

Patients’ characteristics are summarized in Table 1. There were no marked differences in age, sex, body mass index (BMI), Child–Pugh classification, or creatinine clearance among the groups. In addition, no apparent differences were seen in baseline platelet counts among the groups. All the enrolled patients showed APRI >1 and/or FIB4 >3.25 in a post-hoc analysis. Furthermore, 87 % of the enrolled patients (33/38) had 1 of the following complications of liver cirrhosis: edema (2/38), ascites (6/38), esophageal and/or gastric varices (26/38), or HCC (18/38).

**Table 1:** Patient characteristics

		12.5 mg (*N* = 12)	25 mg (*N* = 14)	37.5 mg (*N* = 12)
Age (years)	Median (range)	63.0 (45–81)	58.0 (44–75)	69.5 (48–81)
Sex	Female/male	4/8	4/10	4/8
Body mass index (kg/m^2^)	Mean ± SD	22.6 ± 2.20	25.0 ± 4.15	24.7 ± 4.72
Etiology of liver disease	HCV/HBV/cryptogenic	7/4/1	9/3/2	10/1/2^a^
Child–Pugh classification	A/B	8/4	8/6	7/5
APRI	Mean ± SD	4.3 ± 2.0	4.9 ± 2.8	4.9 ± 2.8
FIB4	Mean ± SD	12.7 ± 3.6	13.8 ± 4.5	16.5 ± 8.2
Baseline platelet count (/μL)	Median (range)	42,500 (36,000–49,000)	38,000 (19,000–48,000)	40,000 (23,000–49,000)
Total bilirubin (mg/dL)	Mean ± SD	1.51 ± 1.19	1.53 ± 0.62	1.27 ± 0.52
Creatinine (mg/dL)	Mean ± SD	0.70 ± 0.22	0.72 ± 0.16	0.83 ± 0.26
Creatinine clearance (mL/min)	Mean ± SD	93.5 ± 29.7	106.1 ± 34.9	84.2 ± 40.4

*HBV* hepatitis B virus, *HCV* hepatitis C virus, *SD* standard deviation, *APRI* aspartate aminotransferase-to-platelet ratio index

^a^One patient in the 37.5 mg group was infected with both HCV and HBV

### Pharmacokinetics

**Fig. 2:**
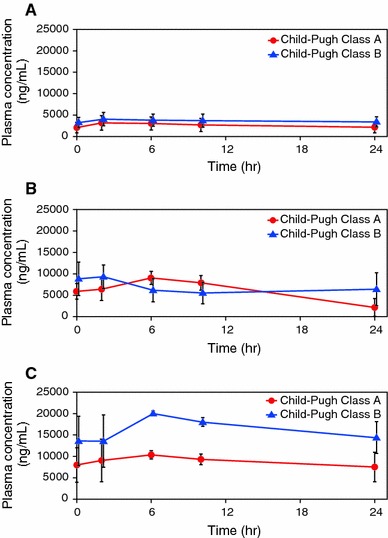
Plasma eltrombopag concentration stratified by Child–Pugh class in the 12.5 mg (**a**), 25 mg (**b**), and 37.5 mg groups (**c**). One patient in the 12.5 mg group with Child–Pugh class A and another patient in the 37.5 mg group with Child–Pugh class B were excluded from summary statistics of plasma concentration, because both patients had used a cation-containing antacid, which affects the exposure of eltrombopag. Data are expressed as means ± SD

The geometric mean of *C*_max_ in the 12.5 mg group was 3,413 ng/mL (95 % CI 2,549–4,570) at approximately 3.4 h after administration, and the geometric mean of the AUC (0–24) was 65,236 ng h/mL (95 % CI 46,748–91,035) (Table 2). There was no apparent difference in the mean plasma eltrombopag concentration stratified by Child–Pugh class in the 12.5 and 25 mg groups (Fig. 2a, b). However, in the 37.5 mg group a higher mean plasma concentration of eltrombopag was observed in patients with Child–Pugh class B compared with patients with Child–Pugh class A (Fig. 2c).

**Table 2:** Pharmacokinetic parameters (12.5 mg eltrombopag group, log-transformed data)

	*N*	*n* ^a^	Geom. mean	95 % CI of geom. mean		SD logs	%CVb
				Lower	Upper		
*C*_max_ (ng/mL)	12	11	3,413	2,549	4,570	0.4345	45.6
*T*_max_ (h)	12	11	3.44	2.459	4.823	0.5012	53.4
AUC(0–*t*) (ng h/mL)	12	11	65,244	46,617	91,314	0.5004	53.3
AUC(0–24) (ng h/mL)	12	11	65,236	46,748	91,035	0.4960	52.8

*CI* confidence interval, *Geom. mean* geometric mean, *CVb* between-subject coefficient of variance, *C*_*max*_ maximum plasma concentration, *T*_*max*_ time to maximum plasma concentration

^a^One patient was excluded from the summary statistics of pharmacokinetic parameters because the patient had used a cation-containing antacid, which affects the exposure of eltrombopag

### Efficacy

#### Primary endpoint

**Fig. 3:**
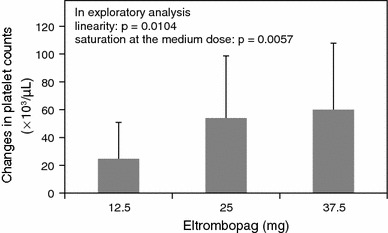
Changes from baseline in platelet counts at week 2. Exploratory analyses were conducted to detect a dose response and trend, using the changes from baseline in platelet counts at week 2. These data were analyzed using analysis of covariance (ANCOVA) with baseline platelet counts as a covariate, using contrast methods for the following dose response patterns: linearity in 3 doses [contrast of 12.5, 25 and 37.5 mg: −1 0 1], saturation at the medium dose (25 mg) [contrast: −2 1 1], and onset of response at the high dose (37.5 mg) [contrast: −1 −1 2]. No adjustment for multiplicity was made. Data are expressed as means + SD

*Changes from baseline in platelet counts at week 2* The mean increases from baseline in platelet counts at week 2 were 24,800/μL (95 % CI 8,200–41,400), 54,000/μL (95 % CI 28,200–79,800), and 60,000/μL (95 % CI 29,300–90,700) in the 12.5, 25, and 37.5 mg groups, respectively (Fig. 3). An exploratory analysis showed statistically significant linearity in 3 doses (*p* = 0.0104) and saturation at the medium dose (*p* = 0.0057) at the 5 % significance level.

#### Secondary endpoints

*Response rate to eltrombopag and effects of an additional 1-week treatment* There were 3 (25 %), 6 (42.9 %), and 7 (58.3 %) patients in the 12.5, 25, and 37.5 mg groups, respectively, who responded (achieved platelet counts of ≥80,000/μL) to eltrombopag at week 2. Six patients in the 25 mg group and 2 patients in the 37.5 mg group with platelet counts of <80,000/μL at week 2 received an additional 1 week of treatment. Of these patients, 3 in the 25 mg group and 1 in the 37.5 mg group responded to the additional week of treatment. Platelet counts in these 4 patients increased to approximately 70,000/μL (range 69,000–74,000) by week 2.

**Fig. 4:**
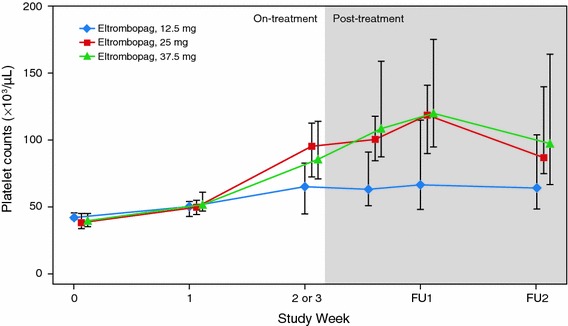
Median platelet counts after treatment with eltrombopag. Platelet counts at either week 2 or 3, or at the end of treatment with eltrombopag. Platelet counts after the end of treatment include the values after invasive procedures or platelet transfusions. Data are expressed as medians with interquartile ranges (IQRs). *FU* follow up

*Median platelet counts after administration of eltrombopag* The median platelet count in the 12.5 mg group increased from 42,500/μL [inter-quartile range (IQR) 40,500–45,500] at baseline to 66,000/μL (IQR, 45,000–83,000) at week 2 and remained at the same level for 2 weeks post-treatment. In contrast, in the 25 and 37.5 mg groups, the median platelet counts increased to 73,000/μL (IQR, 69,000–110,000) and 81,500/μL (IQR, 69,500–114,000), respectively, by week 2. At 1-week post-treatment, the median platelet counts peaked at 119,000/μL (IQR, 90,000–141,000) and 120,000/μL (IQR, 95,500–175,500) in the 25 and 37.5 mg groups, respectively, and remained at >80,000/μL for 1 week thereafter (Fig. 4). The median increases from baseline in platelet counts at week 2 for Child–Pugh class A and B, respectively, were 11,000/μL (range, −9,000 to 83,000) and 28,000 (range, 17,000–30,000) in the 12.5 mg group; 38,500/μL (range, 12,000–100,000) and 50,000 (range, 19,000–187,000) in the 25 mg group; and 46,000/μL (range, 8,000–193,000) and 50,000 (range, 20,000–91,000) in the 37.5 mg group.

### Safety

The incidences of adverse events (AEs) of any grade during the study were 50 % (6/12), 50 % (7/14), and 75 % (9/12) in the 12.5, 25, and 37.5 mg groups, respectively (Table 3). Most AEs reported were grade 1 or 2 in severity. Back pain, pyrexia, and postoperative fever were the most common AEs, and they occurred mostly after invasive procedures. No grade 3 or higher AEs occurred during the treatment period. No subject discontinued eltrombopag because of AEs or platelet counts of >200,000/μL during the study.

**Table 3:** Adverse events (AEs) observed during the study

	12.5 mg (*N* = 12)	25 mg (*N* = 14)	37.5 mg (*N* = 12)
Adverse events with ≥2 patients in any group, *n* (%)	6 (50 %)	7 (50 %)	9 (75 %)
Back pain	1 (8 %)	0	4 (33 %)
Pyrexia	0	3 (21 %)	2 (17 %)
Postoperative fever	3 (25 %)	0	2 (17 %)
Pleural effusion	2 (17 %)	0	2 (17 %)
Abdominal distension	1 (8 %)	0	2 (17 %)
Ascites	1 (8 %)	0	2 (17 %)
Procedural pain	2 (17 %)	0	1 (8 %)
ALT increased	2 (17 %)	1 (7 %)	0
AST increased	2 (17 %)	1 (7 %)	0
All grade 3 or 4 adverse events, *n* (%)	0	0	1 (8 %)
All drug-related adverse events, *n* (%)	1 (8 %)	4 (29 %)	4 (33 %)
Diarrhea	0	1 (7 %)	1 (8 %)
Renal impairment	0	1 (7 %)	1 (8 %)
Abdominal distension	0	0	1 (8 %)
Abdominal pain	0	0	1 (8 %)
Back pain	0	0	1 (8 %)
Eosinophilia	0	0	1 (8 %)
Eosinophil count increased	0	0	1 (8 %)
Anorexia	0	0	1 (8 %)
Pleural effusion	0	0	1 (8 %)
Pain in extremity	0	1 (7 %)	0
Vomiting	0	1 (7 %)	0
Urinary tract infection	0	1 (7 %)	0
Supraventricular extrasystoles	1 (8 %)	0	0
Serious adverse events, *n* (%)	0	0	2 (17 %)
Ascites	0	0	1 (8 %)
Pleural effusion	0	0	1^a^ (8 %)
Portal vein thrombosis	0	0	1^a^ (8 %)
Death, *n* (%)	0	0	1^b^ (8 %)

The severity of adverse events was graded using the Division of AIDS Table for Grading the Severity of Adult and Pediatric Adverse Events (version 1.9, dated December 2004). The data include AEs seen on study plus all drug-related AEs

*ALT* alanine aminotransferase, *AST* aspartate aminotransferase

^a^One patient experienced pleural effusion and portal vein thrombosis 22 days post-treatment

^b^The death occurred 149 days after the end of treatment with eltrombopag

Drug-related AEs occurred in 8 % (1/12), 29 % (4/14), and 33 % (4/12) of patients in the 12.5, 25, and 37.5 mg groups, respectively (Table 3). No drug-related serious adverse events (SAEs) were seen in either the 12.5 or 25 mg groups; however, 2 patients in the 37.5 mg group experienced drug-related SAEs (Table 3).

### Drug-related SAEs

#### SAE#1; worsening pleural effusion and development of portal vein thrombosis

A 63-year-old female cirrhotic patient with HCC, esophageal varices, and pleural effusion was administered 37.5 mg of eltrombopag daily for 14 days. Her platelet count increased from 36,000 to 127,000/μL and there were no AEs during eltrombopag administration. On day 23, the patient underwent partial splenic embolization. On day 35, grade 3 worsening pleural effusion and grade 3 portal vein thrombosis were seen. Platelet counts were 197,000 and 271,000/μL on days 22 and 35, respectively. With conservative therapy, the pleural effusion and portal vein thrombosis improved, on days 77 and 140, respectively.

#### SAE#2; worsening ascites

An 81-year-old female cirrhotic patient with HCC and ascites was administered 37.5 mg of eltrombopag daily for 14 days. Her platelet count increased from 49,000 to 242,000/μL. Although no thrombus was observed on computed tomography (CT) images, grade 2 worsening ascites was seen on day 11. The ascites was refractory to diuretic agents and an albumin preparation and was found to be chylous on day 57. Platelet counts were 87,000 and 197,000/μL on days 9 and 57, respectively. The patient developed cachexia and renal failure, and died on day 163 (149 days after the end of eltrombopag treatment).

### Effects of pretreatment with eltrombopag on the prevalence of perioperative bleeding and platelet transfusions

Of the 38 patients who received eltrombopag, 6 patients underwent a total of 7 invasive procedures with bleeding risk after the end of treatment with eltrombopag. RFA was the most common procedure during the study (Table 4). Five of these patients had platelet counts of >80,000/μL prior to undergoing their invasive procedures, and most of the procedures were safely performed without platelet transfusions (Table 4).

**Table 4:** Effects of pretreatment with eltrombopag on the prevalence of perioperative bleeding and platelet transfusions

Groups	Case number	Procedure	Bleeding	Platelet transfusion	Days after end of treatment	Platelet count (/μL)		
						Baseline	Pre-procedure	Post-procedure
12.5 mg	11	Partial hepatectomy, splenectomy, cholecystectomy	Yes	Yes^a^	8	42,000	46,000	194,000
	31	Radiofrequency ablation	No	No	3	43,000	173,000	178,000
25 mg	5	Radiofrequency ablation	No	No	13	48,000	217,000	152,000
	6	Tooth extraction	No	No	9	45,000	387,000	382,000
		Tooth extraction	No	No	13	45,000	387,000	382,000
	32	Liver biopsy	No	No	1	46,000	146,000	142,000
37.5 mg	51	Radiofrequency ablation	No	No	6	37,000	126,000	183,000

^a^Platelet transfusion was performed prior to invasive procedures

## Discussion

This study demonstrated that significant increases in platelet counts could be achieved by 2-week administration of eltrombopag to Japanese patients with CLD and thrombocytopenia. Our results also show that a maximum of 25 mg of eltrombopag, a lower dose than that typically used in Caucasian patients, can be recommended for Japanese patients with CLD and thrombocytopenia.

In this phase II study, we investigated the pharmacokinetics of eltrombopag in Japanese patients with CLD and thrombocytopenia. An inter-ethnic difference in the pharmacokinetics of eltrombopag has been observed between East Asian and non-Asian patients with ITP, as well as between East Asian and non-Asian healthy volunteers [21]; in our study, the AUC_0–*τ*_ in Japanese patients receiving 37.5 mg of eltrombopag once daily was estimated to be 236 μg h/mL, which is similar to that seen in non-East Asian patients with CLD receiving 75 mg once daily in a previous study [26]. Although the mechanisms underlying an inter-ethnic difference in the pharmacokinetics of eltrombopag remain unclear, a common difference between East Asian and Caucasian ethnic groups is body weight [27]. Because the clearance of eltrombopag increased with body weight [21] and because the body weight of East Asian patients is lower than that of Caucasian patients in general, body weight differences could account for the differences in serum levels of eltrombopag seen between the two groups. A pharmacogenetic study has also been performed to investigate a relationship between gene polymorphisms and inter-ethnic differences; however, the responsible polymorphism has not been identified (data not shown). Eltrombopag is a substrate of several drug-metabolizing enzymes, including cytochrome P450 (CYP) 1A2, CYP2C8, uridine diphosphate-glucuronosyltransferase (UGT) 1A1, and UGT1A3, and the agent is also a substrate of breast cancer resistance protein [27]. Because the activities of these enzymes are known to have inter-ethnic differences [28, 29], it is possible that multiple factors, including genetic differences in metabolizing enzymes and transporters, may be involved in the observed difference [27].

In the present study, platelet counts continued to increase 1 week post-treatment and gradually decreased thereafter. This finding is similar to that seen in another study of eltrombopag in patients with CLD and thrombocytopenia; in contrast, in several studies of eltrombopag in chronic ITP, platelet counts began to decrease at 1 week post-treatment and returned to baseline levels within 2 weeks [19, 30]. Although the reason is unclear, 2 possibilities may account for this difference. First, plasma eltrombopag concentrations in patients with CLD are higher than those in patients with ITP [21, 26], and therefore the differences in exposure between these 2 diseases may be responsible for changes in platelet counts after treatment with eltrombopag. Second, the difference may be based on the pathogenesis of thrombocytopenia. The main cause of thrombocytopenia in patients with ITP is an autoimmune-mediated active platelet destruction. In patients with CLD, increased blood flow into the spleen secondary to portal hypertension and subsequent passive trapping of platelets in the spleen contribute to thrombocytopenia in this patient population [31, 32].

Most AEs reported in the present study were grade 1 or 2 in severity, and no significant aminotransferase abnormalities were observed. The incidence of drug-related AEs in the 37.5 mg group was somewhat higher compared with the other groups, and drug-related serious events (ascites, increase of pleural effusion, and development of portal vein thrombosis) were seen in 2 patients receiving 37.5 mg of eltrombopag. There was 1 portal vein thrombosis event seen after eltrombopag treatment. Post-hoc analyses of a study of non-Japanese patients with CLD and thrombocytopenia receiving 75 mg of eltrombopag showed that maximum post-baseline platelet counts were associated with the thromboses observed in that study [33]. Thus, thrombogenesis seems to be a factor for the development of the thrombotic events. In addition, it has recently been reported that platelets can amplify inflammation [34], suggesting that platelet-amplified inflammation could be a possible factor for the development of thrombosis. The minimum effective dose of eltrombopag should therefore be used in order to minimize the risk of thromboembolic events, and platelet counts should be closely monitored.

It may be unusual to recommend an optimal dose of eltrombopag in Japanese patients with CLD based only on this study. Nevertheless, we propose that 12.5 or 25 mg can be recommended for Japanese patients with CLD and thrombocytopenia undergoing invasive procedures, based on the following 3 findings: (1) a higher mean concentration of eltrombopag was observed in patients with Child–Pugh class B compared with Child–Pugh class A in the 37.5 mg group (Fig. 2); (2) although the mean AUC_0–τ_ increased with an increase in the eltrombopag dose, the increase in platelet counts seemed to be saturated at 25 mg of eltrombopag (Figs. 3, 4); (3) SAEs were seen in the 37.5 mg group. Although the SAEs may have been due in part to invasive procedures or the natural course of the disease, we could not rule out the possibility of these being drug-related.

In our study, except for 1 patient treated with partial hepatectomy, splenectomy, and cholecystectomy, perioperative bleeding was not seen and platelet transfusions were not required before invasive procedures. These findings suggested that eltrombopag may reduce the necessity for platelet transfusions in patients with CLD undergoing invasive procedures.

These findings demonstrated that a maximum daily dose of 25 mg of eltrombopag administered for 2 weeks was effective and well-tolerated for Japanese patients with CLD. Eltrombopag seems to be an efficacious alternative to platelet transfusions for supporting invasive procedures in patients with CLD and thrombocytopenia, although there might be a risk of treatment-related thrombosis. Further studies will help to establish the appropriate use of eltrombopag for supporting invasive procedures in patients with CLD and thrombocytopenia while investigating the effective prevention of thrombosis.

In conclusion, eltrombopag ameliorated thrombocytopenia in Japanese patients with CLD. A daily dose of 25 mg and 2-week administration is recommended for these patients. Recently, Afdhal et al. [35] reported that patients with HCV who were treated with eltrombopag showed significantly higher sustained virologic response rates following interferon-based therapy compared with patients treated with placebo. Therefore, in addition to its role as a supporting agent for invasive procedures, further studies will be focused on the ability of eltrombopag to initiate and maintain the interferon therapy, and subsequently facilitate an increase in the sustained virologic response rate in patients with thrombocytopenia with chronic HCV infection.

## References

[1] Lu SN, Wang JH, Liu SL, Hung CH, Chen CH, Tung HD (2006). Thrombocytopenia as a surrogate for cirrhosis and a marker for the identification of patients at high-risk for hepatocellular carcinoma. Cancer.

[2] Afdhal N, McHutchison J, Brown R, Jacobson I, Manns M, Poordad F (2008). Thrombocytopenia associated with chronic liver disease. J Hepatol.

[3] Rios R, Sangro B, Herrero I, Quiroga J, Prieto J (2005). The role of thrombopoietin in the thrombocytopenia of patients with liver cirrhosis. Am J Gastroenterol.

[4] British Committee for Standards in Haematology, Blood Transfusion Task Force. (2003). Guidelines for the use of platelet transfusions. Br J Haematol.

[5] Samama CM, Djoudi R, Lecompte T, Nathan-Denizot N, Jean-François F, Agence Française de Sécurité Sanitaire des Produits de Santé expert group (2006). Perioperative platelet transfusion: recommendations of the Agence française de Sécurité Sanitaire des Produits De Santé (AFSSaPS) 2003. Can J Anesth.

[6] Norfolk DR, Ancliffe PJ, Contreras M, Hunt BJ, Machin SJ, Murphy WG (1998). Consensus conference on platelet transfusion, Royal College of Physicians of Edinburgh 27–28 November 1997. Br J Haematol.

[7] Rebulla P (2001). Revisitation of the clinical indications for the transfusion of platelet concentrates. Rev Clin Exp Hematol.

[8] Rebulla P (2001). Platelet transfusion trigger in difficult patients. Transfus Clin Biol.

[9] Eisen GM, Baron TH, Dominitz JA, Faigel DO, Goldstein JL, Johanson JF (2002). Complications of upper GI endoscopy. Gastrointest Endosc.

[10] Kawaguchi T, Kuromatsu R, Ide T, Taniguchi E, Itou M, Sakata M (2009). Thrombocytopenia, an important interfering factor of antiviral therapy and hepatocellular carcinoma treatment for chronic liver diseases. Kurume Med J.

[11] Eder AF, Chambers LA (2007). Noninfectious complications of blood transfusions. Arch Pathol Lab Med.

[12] Wilhelm D, Klouche M, Fiebelkorn A, Görg S, Klüter H, Kirchner H (1993). Non-haemolytic transfusion reactions after platelet substitution. Lancet.

[13] Vamvakas EC (2004). Platelet transfusion and adverse outcomes. Lancet.

[14] Okabayashi T, Hanazaki K (2008). Overwhelming postsplenectomy infection syndrome in adults—a clinically preventable disease. World J Gastroenterol.

[15] Stasi R, Evangelista ML, Amadori S (2008). Novel thrombopoietic agents a review of their use in idiopathic thrombocytopenic purpura. Drugs.

[16] Matthys G, Park JW, McGuire S, Wire MB, Bowen C, Williams D, et al. Clinical pharmacokinetics, platelet response, and safety of eltrombopag at supratherapeutic doses of up to 200 mg once daily in healthy volunteers. J Clin Pharmacol. 2011;51(3):301–8 (Epub 2010 Apr 23). 10.1177/0091270010368677 20418510

[17] Bussel JB, Cheng G, Saleh MN, Psaila B, Kovaleva L, Meddeb B (2007). Eltrombopag for the treatment of chronic idiopathic thrombocytopenic purpura. N Engl J Med.

[18] McHutchison JG, Dusheiko G, Shiffman ML, Rodriguez-Torres M, Sigal S, Bourliere M (2007). Eltrombopag for thrombocytopenia in patients with cirrhosis associated with hepatitis C. N Engl J Med.

[19] Cheng G, Saleh MN, Marcher C, Vasey S, Mayer B, Aivado M (2011). Eltrombopag for management of chronic immune thrombocytopenia (RAISE): a 6-month, randomized, phase 3 study. Lancet.

[20] Bauman JW, Vincent CT, Peng B, Wire MB, Williams DD, Park JW. Effect of hepatic or renal impairment on eltrombopag pharmacokinetics. J Clin Pharmacol. 2011;51:739–50. 10.1177/0091270010372106 20663991

[21] Gibiansky E, Zhang J, Williams D, Wang Z, Ouellet D (2011). Population pharmacokinetics of eltrombopag in healthy subjects and patients with chronic idiopathic thrombocytopenic purpura. J Clin Pharmacol.

[22] Heddle NM, Cook RJ, Tinmouth A, Kouroukis CT, Hervig T, Klapper E (2009). A randomized controlled trial comparing standard- and low-dose strategies for transfusion of platelets (SToP) to patients with thrombocytopenia. Blood.

[23] Wai CT, Greenson JK, Fontana RJ, Kalbfleisch JD, Marrero JA, Conjeevaram HS (2003). A simple noninvasive index can predict both significant fibrosis and cirrhosis in patients with chronic hepatitis C. Hepatology.

[24] The Japanese Society of Gastroenterology, editor. Liver cirrhosis practical guideline. Tokyo: Nankodo Co. Ltd.; 2009.

[25] Drinka PJ, Langer E (1989). The Cockroft–Gault formula. J Am Geriatr Soc.

[26] Farrell C, Hayes S, Giannini EG, Afdhal NH, Tayyab GN, Mohsin A (2010). Gender, race, and severity of liver disease influence eltrombopag exposure in thrombocytopenic patients with chronic liver disease. Hepatology.

[27] Shida Y, Takahashi N, Nohda S, Hirama T (2011). Pharmacokinetics and pharmacodynamics of eltrombopag in healthy Japanese males. Jpn J Clin Pharmacol Ther..

[28] Zhang A, Xing Q, Qin S, Du J, Wang L, Yu L (2007). Intra-ethnic differences in genetic variants of the UGT-glucuronosyltransferase 1A1 gene in Chinese populations. Pharmacogenomics J.

[29] Mizutani T (2003). PM frequencies of major CYPs in Asians and Caucasians. Drug Metab Rev.

[30] Afdhal N, Giannini E, Tayyab GN, Mohsin A, Lee JW, Andriulli A, et al. Eltrombopag in chronic liver disease patients with thrombocytopenia undergoing an elective procedure: results from ELEVATE, a randomized clinical trial. J Hepatol. 2010;52:S460 (Abstr 1185).

[31] Cines DB, Blanchette V (2002). Immune thromocytopenic purpura. N Engl J Med.

[32] Giannini EG, Savarino V (2008). Thrombocytopenia in liver disease. Curr Opin Hematol.

[33] Giannini EG, Afdal NH, Campbell FM, Blackman NJ, Shi W, Hyde DK, et al. Exploratory analyses of predictors of thrombotic events in the ELEVATE study. Hepatology. 2010;52:1071A (Abstr 1569).

[34] Boilard E, Nigrovic PA, Larabee K, Watts GFM, Ciblyn JS, Weinblatte ME (2010). Platelets amplify inflammation in arthritis via collagen-dependent microparticle production. Science.

[35] Afdhal N, Dusheiko G, Giannini EG, Chen P, Han K, Moshin A, et al. Final results of ENABLE 1, a phase 3, multicenter study of eltrombopag as an adjunct for antiviral treatment of hepatitis C virus-related chronic liver disease associated with thrombocytopenia. Hepatology. 2011;54:1427A–28A (LB-3 Abstract form).

